# Community engagement as a foundation for implementation research for group psilocybin assisted therapy in New Mexico

**DOI:** 10.3389/fpubh.2026.1845943

**Published:** 2026-06-17

**Authors:** Lawrence Leeman, Maya Armstrong, Dara Menashi, Hanifa Nayo-Washington, Elliot Marseille, Janus Herrera, Crystal Romero, Janet Page-Reeves

**Affiliations:** 1 Department of Family and Community Medicine, School of Medicine, University of New Mexico, Albuquerque, NM, United States; 2 Psychedelic Mental Health Access Alliance, Boston, MA, United States; 3 Psychedelic Mental Health Access Alliance, Santa Fe, NM, United States; 4 Collaborative for the Economics of Psychedelics, University of California Berkeley, Berkeley, CA, United States; 5 Health Equity Council, Albuquerque, NM, United States

**Keywords:** group psychedelic therapy, peer facilitators, psilocybin, PTSD, veterans, women survivors of sexual violence

## Abstract

Informed by a community engagement process, we have developed a pragmatic, open-label, hybrid feasibility-implementation study of Group Psilocybin-Assisted Therapy (GPAT) for post-traumatic stress disorder (PTSD). As psychedelic-assisted therapies begin to enter the broader mental health arena, engaging communities in the design of research and care models is essential to ensuring that those most impacted have access to—and trust in—the treatments being developed. Guided by the principle of “nothing about us without us,” the proposed study was designed from the outset to be community-informed and co-designed. We established a tripartite collaborative partnership among researchers from the University of New Mexico (UNM) Department of Family & Community Medicine (DFCM) and the UNM Office for Community Health (OCH), the Bernalillo County Health Equity Council, and the national Psychedelic Mental Health Access (PMHA) Alliance. The protocol for the FDA-approved study uses a group therapy model incorporating peer facilitators with shared affinity with the participant group. There will be six groups of six participants, including veterans/first responders and women survivors of sexual violence. The study is aligned with the New Mexico Therapeutic Psilocybin Program in its focus on access and equity, as well as the use of state-regulated whole psilocybin mushrooms rather than synthesized psilocybin. Each group will participate in two sessions using psilocybin mushroom-infused chocolates containing 20 mg and 30 mg of psilocybin, along with both group and individual therapy sessions. Outcomes will include safety and feasibility measures, as well as standard measures of PTSD, including the Clinician-Administered PTSD Scale for DSM-5 (CAPS-5) and the PTSD Checklist for DSM-5 (PCL-5). An extended integration model provides longer-term support to participants both within and beyond the clinical trial. A barrier to scaling psychedelic-assisted therapy is understanding what it actually costs to deliver a safe, comprehensive model of care, particularly for populations facing structural barriers to access. The University of California, Berkeley Collaborative for the Economics of Psychedelics (CEP) will conduct a prospective micro-costing study in parallel with GPAT. The analysis will capture delivery costs for community engagement, the group psilocybin therapy model, and the post-clinical protocol extended integration program.

**Study protocol registration:**

https://clinicaltrials.gov/study/NCT07506395.

## Introduction

Given the growing interest in psychedelic-assisted therapies (PATs), there is a great need to expand the scientific literature through well-designed clinical trials. As most psychedelic compounds are still classified as Schedule I by the United States Drug Enforcement Administration (DEA), such trials require approval by the Food and Drug Administration (FDA) in the form of an Investigational New Drug application (IND). In recent years, most clinical trials of psychedelic therapies have been sponsored by pharmaceutical companies focused on obtaining FDA approval for a proprietary compound. The goal of FDA approval has led to a focus on individual rather than group treatment, stringent exclusion criteria that do not reflect “real world” populations, and patentable compounds rather than botanical sources. To date, multiple psychedelic compounds have been granted FDA “breakthrough” status, which allows accelerated approval for testing of treatments with evidence of potential improvement over existing therapies. However, despite the interest in the emerging evidence of impact for psychedelic agents and PAT approaches, three dimensions of the current approach in PAT clinical trial research have received insufficient attention in the literature.

The first is the need for exploration of care models that expand the narrow focus on individual health outcomes to include patient and community acceptability. Demand for mental health care far outstrips supply, with disproportionate burdens falling on young people, low-income communities, communities of color, and Tribal Nations. In the United States, mental-health conditions now affect an estimated one in five adults annually, with especially high rates among Black, Indigenous communities, and LGBTQ+ communities, young people, those living in poverty, individuals in the postpartum period (1).

The second is the need for sustainable health care delivery models with equitable access to contribute to economic viability. Inequities in mental health services across the United States that leave people without access to care were estimated to cost the country nearly half a trillion dollars in avoidable expenses in 2025—and could escalate to $1.3 trillion by 2040 if left unaddressed (2). To move the needle on these alarming statistics, PAT research needs to ensure that the voices of those most impacted by these conditions are included in the development and the design of PAT care models. Given the promise of PAT in addressing mental and behavioral health issues that disproportionately impact minoritized individuals and those from low-income households, lack of inclusion in clinical trial studies is an egregious gap in the science underpinning the development of PAT treatment.

The third aspect of PAT research that has not received sufficient attention is that three states now have FDA-regulated models allowing therapeutic use of whole *Psilocybe cubensis* mushroom use. Synthesized psilocybin and whole psilocybin mushrooms may have distinct effects. As such, there is an urgent need for studies that use whole psilocybin mushrooms rather than synthesized psilocybin.

At the University of New Mexico (UNM), we have designed a PAT clinical trial that addresses all three of these gaps in the literature. We have received FDA approval for a clinical trial to test a group therapy approach using whole psilocybin mushrooms in chocolate for the management of PTSD. Our study incorporates community engagement in the design and operation of the research, and we hypothesize that our group therapy design will offer a cost-effective delivery model. Our study integrates:

- Group-format psilocybin therapy
- Pragmatic inclusion and exclusion criteria
- Trained peer facilitators with lived experience and shared affinity in addition to licensed therapists
- Use of whole mushrooms in alignment with the New Mexico State regulated Medical Psilocybin program
- An extended integration model for longer-term support to participants including the post-clinical protocol the *Circle of Care* program
- Concurrent cost modeling assessment

Here, we describe our program model, including the clinical trial and the adjacent community engagement and cost modeling processes.

## Access and equity perspective

Psychedelic-assisted therapy is supported by a growing evidence base showing rapid and often sustained improvements in difficult-to-treat conditions, such as PTSD, depression, and substance use disorders (3–5). Recognizing the critical nature of our mental and behavioral problems that have reached crisis levels across the country, we believe it is urgent to design effective, scalable interventions, coupled with culturally competent preparation and integration support, and to make these widely accessible to those most in need. Without direct efforts to ensure these promising interventions have broad reach, we are in danger of replicating the current system of unequal access to care that we see in our current health care system. Unless we consciously develop PAT to have an equity focus from the beginning, those who suffer the most from mental health issues are the least likely to be able to access these treatments.

New Mexico is a state known for the majestic beauty of its land, multicultural populations, strong and resilient communities, and cultural traditions with deep roots. It also has high levels of poverty, violence, and intergenerational trauma, which have contributed to the devastation caused by the opioid epidemic and the associated cycles of despair. Tragically high rates of trauma and PTSD inspired New Mexican legislators to pass the New Mexico Medical Psilocybin Act, signed into law in April 2025. The Act currently names four conditions eligible for PAT: PTSD, treatment-resistant depression, substance use disorders, and end-of-life existential distress. Furthermore, it emphasizes equitable access to PAT and supports the creation of an evidence base through research.

The University of New Mexico (UNM) Research program for PAT began a collaboration with the Psychedelic Mental Health Access Alliance (PMHA Alliance), a group founded in 2023 in response to the growing demand for PAT and the deep, ongoing inequities in access to effective mental health care. PMHA Alliance is dedicated to developing Medicaid-fundable, community-informed psychedelic therapy models of care that are ethical, inclusive, and effective. These models of care must be affordable, leverage existing available workforce, and integrate into the existing community-based services and organizations serving people from marginalized communities. To provide the robust evidence that Medicaid decision makers need to cover the cost of PAT, these models must be rigorously tested in a real-world setting with a population that is similar to those on Medicaid.

Informed by a community engagement process, we have developed a pragmatic, open-label, hybrid feasibility-implementation study of group-format psilocybin-assisted therapy (GPAT) that uses a whole-mushroom product (in chocolate) for people with PTSD. As of April, 2026, the study has received philanthropic and state funding, FDA IND approval and UNM Human Research Protections Office (HRPO) protocol approval, ClinicalTrials.gov registration and participant screening is anticipated to start in May 2026 (6).

## Enhancing generalizability and access through pragmatic study using a hybrid implementation/effectiveness approach

Our project and clinical study developed in response to requests from patients and community members for access to PAT for trauma and addiction as well as legislature-driven interest in exploring novel approaches to treating major sources of disability in New Mexico, which is largely rural and underserved. It was informed by a process of community engagement, which could be adapted to a wide variety of communities and populations. Our dedication to equitable access is reflected in the use of pragmatic criteria to reduce barriers to participation. Not only is there an urgent need to move research forward to identify safe and effective treatments for PTSD and other conditions but also to work toward efficient implementation strategies that promote equity of access to promising potentially life-saving therapies by moving research into use in the community.

Implementation science focuses on how to more rapidly, efficiently, and sustainably translate evidence into practice. In the typical research pipeline, which is linear and stepwise, there often are significant delays before new innovations are implemented broadly (7). Hybrid effectiveness-implementation studies contribute to accelerating the translation of evidence into practice for the benefit of safer and better healthcare services (8), resulting in more rapid translational gains in uptake of new clinical interventions, more effective implementation strategies, and more useful information for researchers and decision makers (9, 10). There is a growing recognition of the need for PAT studies that reduce barriers to implementation by (1) incorporating pragmatic yet safe inclusion and exclusion criteria, (2) leveraging group therapy approaches, (3) addressing issues of access and cost, and (4) using hybrid effectiveness designs (11, 12).

High-quality, double-blind, randomized controlled trials (RCTs) of psychedelic and other novel therapies generally exclude participants with multiple mental health diagnoses or substance use disorders (SUDs). However, a high proportion of people with PTSD have major depression, and many self-medicate with a variety of substances (13, 14). However, clinical trial research often artificially limits criteria for participation to an unrealistically narrow range that excludes people in the real world who have acute need for treatment. Because the FDA requires a restricted focus regarding the treatment indication under study, Phase 2 and Phase 3 clinical trials required for drug approval include a long list of exclusion criteria. Although this approach produces “cleaner” data, it limits the generalizability of research findings and leaves important gaps in understanding real-world safety and efficacy.

As discussed below in the section describing participants, we have consciously designed this study to be culturally and contextually situated and patient centered. We use inclusion and exclusion criteria that allow the intervention to be accessible to a high proportion of people with PTSD while maintaining participant safety. For example, we do not exclude participants with major depression. Similarly, the use of substances including marijuana, alcohol, sedatives, stimulants, and opiates will not necessarily exclude a candidate from participation in the study. The individual will instead be screened for criteria for an active substance use disorder (and referred to recovery services, if applicable) and if not excluded for this reason, asked to abstain from use for a designated period of time before and after the psilocybin sessions. This form of pragmatic psychedelic research is in alignment with a recent commentary by Robin Carhart-Harris, a leading neuroscientist and researcher in the field of psychedelic medicine, who advocates for studies that incorporate “real-world” situations, such as inclusion of participants with multiple diagnoses and the use of research methodologies other than randomized controlled trials (15).

Moreover, by including a combination of individual and group care therapy sessions we have developed a model that has more potential to be sustainable in under-resourced communities. Per guidance from Pearson et al. (16) for conducting feasibility studies for implementation trials, our pragmatic study will assess the feasibility of community-based implementation of GPAT as a model for dissemination to other communities. The current landscape of psychedelic medicine consists largely of costly, private enterprise that poses a barrier to treatment and healing, further exacerbating social inequities. The intentional propagation of best clinical practices for equity in access to effective care is one way to improve patient outcomes across socioeconomic strata.

## Rooted in community engagement

Research consistently demonstrates that community engagement improves the cultural relevance, fit, and adoption of health interventions, and that it establishes the mutual trust essential to both data quality and sustained community participation (17). This is especially true for communities that carry distinct cultural protocols, spiritual frameworks, and histories of institutional harm. As PAT moves rapidly toward broader clinical application, engaging communities in the design of research and care models is essential to ensuring that those most impacted have access to — and trust in — the treatments being developed. Guided by the principle of “nothing about us without us”, the proposed study was designed from the outset to be community-informed and co-designed. Toward this end, we established a tripartite collaborative partnership among researchers from the University of New Mexico (UNM) Department of Family & Community Medicine (DFCM) and the UNM Office for Community Health (OCH), the Bernalillo County Health Equity Council, and the national Psychedelic Mental Health Access (PMHA) Alliance — each bringing distinct and complementary expertise to this work.

The UNM Health Sciences Center (UNM HSC) is internationally known for its community-engaged and community-based research approaches, and the UNM OCH is internationally recognized as a leader in research focusing on scientifically significant contextual factors that influence health. The UNM HSC, and especially the UNM OCH, have led work to shift public health efforts to a community health focus, which recognizes the key role of context in the medical care system. In addition, UNM HSC has a history of research in the use of psychedelic medicines including research studies of psilocybin in treating alcohol use disorder. For the purposes of this project, we are leveraging work by UNM researchers in the medical dimensions of psychedelic therapies and our experience using Community-Driven, Community-Engaged Research (CD-CEnR) practices. To launch this project, we partnered with two organizations, one local and one national. The Health Equity Council (HEC) is a health coalition in Bernalillo County, New Mexico with deep local roots and expertise in the creation of culturally relevant health resources and fostering community-driven solutions. The Psychedelic Medical Health Access Alliance (PMHA Alliance) is a national coalition dedicated to co-designing psychedelic-assisted therapy care delivery models that unite partners across public, private, clinical, and community sectors — with particular attention to historically marginalized communities and translating and scaling learnings into strategies for broader national application.

Given the complex medical, regulatory, legal, economic, cultural, and social dimensions of PAT research, and further complexity introduced by how they become integrated in a research context, most PAT studies have been designed and conducted using academic and clinically centered approaches. Incorporating community engagement to inform study design or in the operational dynamic of the research has not been the norm. As such, throughout our process for developing the GPAT study, community engagement with diverse stakeholders provides capacity to co-develop a culturally and contextually situated pilot program to introduce GPAT for the treatment of PTSD in a way that will help address long-standing criticisms about lack of clinical trial diversity, equity, and inclusion (18, 19). Therefore, our work is unique in that it fills these gaps. In the PAT research space, we are positioned to break the mold of research “on” participants from communities under-represented in research who are disproportionately impacted by SUD by creating mechanisms for research in partnership “with” them.

The intellectual backbone of our community-engagement approach is Trickett’s model of culturally and contextually situated research (20). Trickett proposes that an intervention needs to be integrated “into the local expression of culture as reflected in the multiple levels of the ecological context” (20). It is not enough to “tailor” an intervention with superficial trappings of language, culture, or images. Instead, Trickett proposes that an intervention needs to be properly “situated” within the constructs of the culture and in relation to the context in which people live their everyday lives. To be successful, an intervention needs to find a cultural and contextual “fit.” Rather, an intervention needs to be strategically designed to leverage dimensions of culture and context to enhance intended outcomes. In this way, the intervention not only aligns with contextually relevant factors and cultural values, but culture becomes a vehicle for promoting positive outcomes.

Over the past decade, community engagement in research has moved from something that was considered not only irrelevant but even antithetical to rigorous scientific clinical studies, to something that is now being increasingly embraced by the scientific community and funders. Engaging patients and community members in conceptualizing research questions and co-designing interventions has been shown to improve the scientific quality of research. In our study, community engagement has provided information regarding regional knowledge, attitudes, and practices related to the potential use of PAT to impact PTSD in different populations.

Led by HEC, we used a variety of engagement practices to inform the development of the GPAT model, including an innovative community “Design Studio” event with a post-event survey, plus focus groups and individual interviews. The Design Studio event incorporated pre-session grounding, group dialogue, shared storytelling, trauma-informed questions, and iterative discussion of PAT design considerations—from preparation to integration. The post-session survey collected further reflections and feedback from participants, sharing their thoughts on the cultural relevance, safety, and inclusivity of the engagement process. Focus groups and interviews were targeted to intentionally incorporate attention to the perspectives of patients with different lived experience such as veterans and first responders, or women survivors of sexual violence. Each of these populations has unique PTSD-related needs that must be incorporated to make the GPAT model appropriate.

In addition, because of the important cultural and historical role of psychedelic medicines in Indigenous culture, we have also been intentional to incorporate input and insights from Indigenous and spiritual healers and other individuals from Indigenous communities to help us design our approach to be respectful and to lessen or mitigate cultural appropriation in the way that we implement GPAT. Our intention throughout has been that our community engagement approach is grounded in respect and dignity, and highlights community and patient voices through dialogue. As Kar and Bhugra (21), propose, conscious attention to dignity through a “constellation” of methodological approaches is necessary to address structural and personal harm in the continued creation of mental and behavioral health disparities for minoritized communities.

## Affinity group/peer navigator model: neurological benefits meet social reinforcement

Although the literature on group-format psychedelic therapies in clinical settings is sparse, emerging data suggest feasibility and efficacy in the treatment of disorders related to trauma and/or shame (22–25). In the study by Anderson et al. (22), the group format not only decreased the number of therapist-hours (and therefore cost) but also was associated with a good safety profile and strongly positive outcomes. Although this study did not compare group therapy to individual therapy, the authors were optimistic about “group therapy’s unique capacity to address social isolation, shame, and stigma” (22). A small study using cohorts of four participants in group-format psilocybin-assisted psychotherapy to treat depression in cancer patients with pain demonstrated decreases in depressive symptoms at 2 and 26 weeks (26). A study of major depression in 30 cancer patients using individual psilocybin sessions and group preparation and integration in cohorts of three to four participants demonstrated a sustained response in 80% at 8 weeks (27). Although the topics of enhanced sense of community and the potential for increased efficacy were not discussed in the published data from this study, many of the participants reported that the group format was particularly important (M. Agrawal, personal communication, 6/17/2025). Furthermore, at least one analysis of group-format PAT was shown to be associated with improved access and decreased cost (28).

We hypothesize that leveraging a group-based therapeutic model will reduce costs while enhancing outcomes. Peer support has been shown to increase social connectedness, reduce isolation, and create or enhance the community safety net (29–31). Adding the group structure enhances social support and connectedness, which are seen as important protective factors (32, 33). This approach may not only make treatment more acceptable and financially accessible but also may amplify impact by fostering a sense of community, social support, and connection (22, 34). Facilitator training will ensure skill development in group facilitation and PAT. The value of the group format will be assessed through feedback collected with qualitative interviews at the end of each cohort.

In this pilot study, we hope to build in social support not only related to the study condition and subgroup affiliations but also to the unique and often intense experiences occasioned by psilocybin. By enhancing social connectedness among individuals who normally are marginalized, stigmatized and/or otherwise isolated, group-format PAT may improve therapeutic potential and sustainability (22, 35). We propose that the use of group therapy, specifically organized by affinity group and incorporating peer facilitators will reduce stigma, decrease barriers to initiating treatment, and foster group cohesion. We further propose that the shared identity will enhance trust among participants and facilitators, improving acceptability to group therapy, and that the shared experience of PAT and subsequent integration will strengthen relational support systems, ultimately enhancing efficacy and durability of treatment.

The facilitation team will consist of at least four members: Two licensed providers (one of whom is designated the “lead facilitator”), and two affinity-group peer-facilitators. The two licensed facilitators will each be licensed healthcare providers with professional training, clinical experience in psychotherapy, and licensed to practice independently. All facilitators will have received acceptable training in PAT, trauma-informed care, and group facilitation.

The peer facilitators will have lived experience with PTSD, and 150 h of training in psilocybin-assisted therapy facilitation approved by the state of Colorado or New Mexico or in a nationally approved program. Peer facilitators with lived experience of affinity group-specific trauma are integral to the facilitator team, not only to increase participant comfort but to foster trust, credibility, and psychological safety within the group setting. Beyond reducing perceived differences in social position, peer facilitators serve as a bridge between the clinical environment and participants’ lived realities, supporting engagement across preparation, dosing, and integration phases. Their presence may enhance participants’ willingness to share openly, help contextualize difficult experiences, and support the translation of insights into meaningful changes in daily life. This role is particularly important in affinity groups such as veterans, first responders, and survivors of interpersonal violence, where shared lived experience can significantly influence trust, retention, and overall treatment acceptability.

All licensed and peer facilitators will attend a project-specific 3 days of training that will occur in New Mexico and virtually which will focus on group psychedelic facilitation and trauma-informed care specific to the affinity groups. Licensed therapists based in New Mexico with experience in group therapy and expanded state of consciousness will use a blend of didactic and experiential approaches including group therapy technique, somatic awareness, mindfulness and breathwork for 8–12 h of the training. Additional aspects of the training will include:

- The Heroic Hearts Project (HHP) is a veteran-led group with extensive experience developing GPAT settings for veterans with PTSD. They are developing training programs for therapists and peer facilitators and have created a training guide. HHP will send a highly experienced facilitator to New Mexico to conduct 8–12 h of training directed toward Veteran/First Responder affinity groups, but which will also be beneficial for all the PTSD affinity groups.
- The Lucy Artificial Intelligence psychedelic therapy trainer created by the Fireside project will be used for training using group scenarios specifically designed for the GPAT study (36).
- Chris Stauffer, MD is the director of the Social Neuroscience and Psychotherapy (SNaP) Lab in Portland Oregon. He has developed research studies for GPAT for demoralized patients with HIV and group MDMA Assisted Therapy for veterans with PTSD.
- Legacy providers and Indigenous people from New Mexico with extensive experience in group psychedelic sessions and ceremonies will share their expertise during a training session. New Mexico has many individuals who have guided group psychedelic sessions. This occurs in ceremonies involving a variety of psychedelic substances held by legacy providers. The experience and knowledge of these providers will augment the facilitator group’s training process.
- Virtual Integration: Training and Mentorship for Peer Navigators — Peer navigators receive supplemental training in leading virtual psychedelic integration circles, covering trauma-responsive facilitation, group process management, grounding and narrative integration techniques, and establishing psychologically safe digital environments, with attention to relational, intergenerational, and collective dimensions of trauma.

## Why use whole mushrooms?

Oregon and Colorado have developed state-regulated models for the use of psilocybin mushrooms, with the Colorado program including approval for therapeutic use in the management of mental health conditions. Therapeutic use of psilocybin mushrooms has been indirectly supported by clinical trials using laboratory-synthesized psilocybin. New Mexico’s Medical Psilocybin Act specifies the use of psilocybin mushrooms rather than laboratory-synthesized psilocybin. The state program will specifically cover the use for four conditions: post-traumatic stress disorder, (PTSD), treatment resistant depression, substance use disorders, and end-of-life distress.

Although psilocybin and its active metabolite, psilocin, are considered to be the primary active ingredients in psilocybin mushrooms, there are at least 23 alkaloids including six other tryptamines found in whole mushrooms (37). Although the use of synthesized psilocybin offers the ability to have a standardized product with regard to strength and composition from a drug-delivery perspective, the use of a whole psilocybe cubensis product could be further beneficial due the potential “entourage” effect of the combination of active compounds (38). This has been suggested by a computational framework simulation that incorporated network pharmacology, molecular docking, and molecular dynamics of eight compounds derived from psilocybin mushrooms. The authors concluded that there was a mechanistic rationale supporting the potential of a different and potentially increased beneficial effect of whole psilocybin mushrooms compared to synthesized psilocybin (39).

There is a paucity of blinded clinical evidence comparing synthesized psilocybin to whole mushrooms. In a study in mice that compared synthesized psilocybin to a “full spectrum” psilocybin mushroom extract (PME), findings suggested that PME was associated with increased neuroplasticity compared to synthesized psilocybin (39). And in a very small unblinded qualitative study of individuals using legal psilocybin in various formulations in Canada through the Roots to Thrive program for end-of-life palliative care, some participants reported preference for the whole mushroom fruiting body over the synthetic product. They described the whole mushrooms as being “sacred, alive, unmanipulated by humans and natural” and having a “gentler onset and comedown.” At the peak of the experience, however, the effects for both types of mushroom preparations were described as being similar (40).

As a phase one study, we hope to demonstrate safety of the use of the whole mushroom product in a therapeutic group setting and provide initial data related to efficacy. Ultimately, well-designed trials will be needed for direct comparison of whole-mushroom fruiting body with laboratory-synthesized psilocybin.

## Clinical study protocol

### Research design and overview

**Figure 1:**
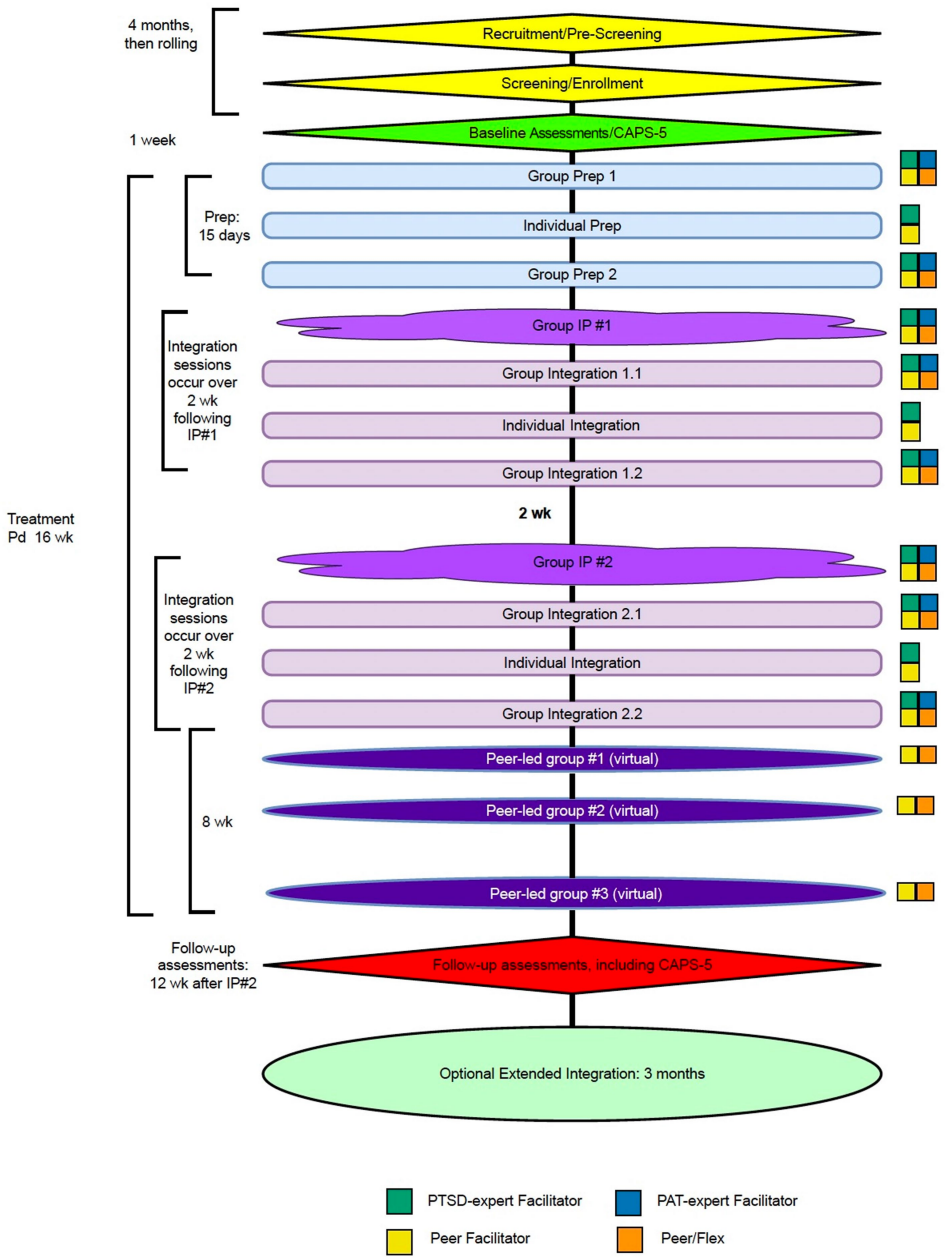
Affinity cohort timeline.

This is a community-informed, pragmatic, open-label, phase-I clinical trial of group-format psilocybin-assisted therapy (GPAT) for individuals with post-traumatic stress disorder (PTSD). Six affinity-based cohorts of six participants each (goal N = 36) will undergo three preparatory sessions, two group-format sessions of GPAT led by two licensed providers and two trained peer-facilitators, and a total of six integration sessions (see Figure 1). As discussed above, we have designed the study to be a low-barrier, pragmatic study with the goal of approximating real-world populations treated in community settings, while also maintaining safety for participants. Psychedelic integration refers to the process by which individuals assimilate the psychological, somatic, and relational effects of psychedelic experiences into their ongoing lives. Bathje, Majeski and Kudowor (41), characterize it as an iterative process of meaning-making that encompasses cognitive, emotional, behavioral, and social dimensions of healing, extending well beyond the acute pharmacological event itself. In appreciation of this perspective and community stakeholder input we are developing an extended integration model that operates on two complementary levels. Within the clinical phase, the therapist-led integration sessions are followed by group sessions facilitated by the cohort’s peer facilitators, ensuring continuity from preparation through integration. Beyond the clinical phase, the Circle of Care provides community-based, participant-directed integration support.

### Participants

We will recruit adult individuals with moderate to severe PTSD, who self-identify as being in at least one of three affinity groups. The two confirmed affinity groups are (1) veterans and first responders and (2) women survivors of sexual violence. The third affinity group has been proposed to be composed of Indigenous people and this plan is in development.

### Objectives

The primary objectives of this study are to assess the safety and feasibility of GPAT for individuals with PTSD, to identify affinity group-specific design components, and to evaluate preliminary effects of the intervention on PTSD severity. Safety outcomes will focus on adverse events, including those of special interest (suicidality, persisting perception disorder). Effects on PTSD will be assessed using changes in the Clinician-Administered PTSD Scale for DSM-5 (CAPS-5) and the PTSD Checklist for DSM-5 (PCL-5) from baseline to final assessment. Secondary objectives are to evaluate the preliminary effects of GPAT on anxiety, depression, disability, and quality of life. Effects of GPAT on chronic pain and substance use are also of interest.

### Setting/environment

Most or all sessions will take place at the Interdisciplinary Substance Use and Brain Injury (ISUBI) Center on the UNM campus. The ISUBI group-therapy room is a simply decorated, comfortable space furnished with a sound system and otherwise modified to accommodate six participants and four facilitators. Two restrooms are nearby. If needed during the dosing sessions, there also is a second space (known as the “apartment room,” which includes a private bathroom), which will be available if a participant needs to leave the larger group temporarily. Should this occur, they will be accompanied by one of the facilitators and joined by a support person to ensure two people are with any participant who is separated from the group.

### Inclusion/exclusion criteria

As a pragmatic design, this study intentionally reduces exclusion criteria, with the goal of approximating real-world populations that would be treated in community settings, while also maintaining safety for participants. The decision to exclude any potential participant will be based on the individual’s ability to adhere to study requirements, their overall physical and psychological stability, and other safety concerns related to the use of psilocybin and the need to abstain from named substances during certain portions of the study.

Potential participants on a stable dose of a selective serotonin reuptake inhibitor (SSRI) antidepressant at the time of screening may be included. Although results from an online retrospective survey suggested that concomitant use of antidepressants may diminish the effects of psilocybin mushrooms (42), other evidence suggests these commonly prescribed drugs do not substantially alter the safety, acute effects, or persisting antidepressant effect of psilocybin (43). Because of insufficient data regarding concomitant use of other antidepressants, non-SSRI antidepressants will need to be tapered and discontinued prior to baseline for an individual to be considered for the study.

### Eligibility and research assessments

A detailed medical and psychiatric history will include all the areas addressed in the inclusion and exclusion criteria (Table 1), including assessment of current medications.

**Table 1:** Inclusion and exclusion criteria.

Inclusion criteria	Exclusion criteria
Age ≥18 years	**Medical**
Diagnosis PTSD with PCL5 ≥ 34	Pregnancy/breastfeeding
Member of one of the defined affinity groups	Poorly controlled diabetes (HbA1c > 8.0%; clinically significant hypoglycemia in the past 6 months)
Not pregnant, planning to become pregnant, or breastfeeding; if able to become pregnant, willing to use reliable form of birth control for the duration of the study	*Cardiovascular*: >140/90 mm Hg; history of myocardial infarction, congestive heart failure, clinically relevant valvular heart disease, or pulmonary hypertension; prolonged QTc
If needed, ability and willingness to taper and discontinue medications that may interfere with the action of psilocybin	*Neurologic*: seizure disorder; neurodegenerative conditions; brain malignancy
Ability to read, speak, and understand English	Unstable thyroid disorder
Ability and willingness to swallow capsules	Severe renal or hepatic impairment
Ability and willingness to consent to the terms of the study	Serious abnormalities of complete blood count or chemistry
	**Psychiatric**
	Active suicidal ideation; history of hospitalization for suicide attempt within the 12 months prior to screening
	Confirmed diagnosis of bipolar I or II disorder, schizophrenia, or other psychotic disorder; first-degree relative with schizophrenia
	Axis-II diagnosis e.g. borderline and sociopathic
	Substance use
	Use of psychedelics resulting in a discrete psychedelic experience in the past 6 months.
	Inability or unwillingness to remain abstinent from cannabis use for 24 h prior to psilocybin dosing session and 12 h after receiving the dose of psilocybin
	Risk for clinically significant acute withdrawal from any substance that would cause safety concern on the day of dosing
	**Other**
	Inability or unwillingness to taper and discontinue contraindicated medications
	Returning to an unsafe environment and/or inadequate social support as determined by during qualitative therapist assessment and use of the MSPSS (46)
	Any other condition, symptom, or other relevant finding that, based on the clinical judgment of trial personnel, would make a participant unsuitable for the trial
	Participation in experimental treatment for PTSD or any research studies within 30 days of screening assessment

Screening procedures include:

- Assessment of vital signs (blood pressure, heart rate, oxygen saturation, and temperature)
- 12-lead electrocardiogram (ECG) and 1-min rhythm strip
- Urine drug screen (UDS) and urine pregnancy test when indicated
- Blood draw for complete blood count (CBC), comprehensive metabolic panel (CMP), and thyroid stimulating hormone (TSH).
- Research assessments (Table 2) will occur at screening, baseline, the conclusion of each medication session, one week after each medication session, and twelve weeks after the second medication session.

### Preparatory sessions

Between baseline evaluation and the first dosing day, there will be three preparation sessions: two group-format sessions and one individual session. In the individual session, at least one of the licensed facilitators and one peer-facilitator will be present. The two group-format preparation sessions will include all participants, two licensed facilitators, and two peer facilitators, if possible, with the understanding that one of the facilitators may need to miss a group session due to scheduling complexities. A fifth person, who may be a licensed therapist, peer facilitator, trainee or a member of the research team will act in a supportive capacity on the dosing day may also be present at the group preparatory sessions.

Minimum total time for the preparation sessions before the first psilocybin medicine session will be 6.0 h. In addition to establishing rapport among participants and facilitators, the following topics will be addressed:

- Orientation to the treatment space and the structure of sessions
- General education about psychedelics and current evidence supporting their role in the management of a variety of mental health conditions
- Discussion of expectations for behavior and conduct, confidentiality, safety, and consent for touch
- Exploration of the diversity of possible experiences while under the influence of psilocybin and strategies for managing any emergent anxiety or other emotions
- Participant goals, intentions, and fears/concerns

### Psilocybin sessions

Two group-format dosing sessions will be scheduled 4 weeks (+/− 1 week) apart, with intervening integration sessions (see next section) (Table 2). On each dosing day, prior to administration of psilocybin, the following exclusion criteria specific to day-of-dosing will be assessed:

- Positive pregnancy status (assessed by urine pregnancy test)
- Urine drug screen (UDS) with unexpected positive results
- Persistently elevated blood pressure (>145/95 mm Hg) or heart rate (>100 bpm)
- Active intoxication with any substance
- Other conditions compromising participant safety during dosing day, as assessed by the on-site physician.

**Table 2:** Schedule of procedures for group experimental session.

Approx time	Procedure or action
08:00	Arrival at site; confirmation of post-procedure plan for ride home and support
08:15	Urine collected for drug screen; assessment of pregnancy status and vital signs
08:30	Review of concomitant medications reviewed; assessment of adverse events
08:45	Eligibility reconfirmed by investigator
09:00	Dosing day orientation: Meet facilitator team in session room, review procedures for the day and discuss participant intentions for session
09:15	Participants encouraged to use restroom
09:30	IMP administered: 20 mg or 30 mg psilocybin (whole mushroom) in chocolate
10:30	Assess blood pressure and heart rate at one hour after receiving IMP and every one to two hours until released to home
14:30–15:30	At approximately six hours after ingestion of the IMP post-ingestion of the M, the participants will be offered the opportunity to enter group-format discussion. Facilitators will use their collective judgment regarding timing. Participants may choose to remain “internal” with eyeshades and headphones if desired.
16:30–17:00	Participant completes post-session measure assessments: MEQ-30, CEQ, EBI, CSSRS, Assess/document full vital signs including BP, heart rate and temperature
17:00–17:30	Assessment of participant readiness for release to home by study physician/PI
17:30–18:00	Review of adverse events by study team

All participants will receive the investigational medicinal product (IMP): 20 mg psilocybin (equivalent, in a whole mushroom chocolate blend) during the first session and 30 mg psilocybin (equivalent, in a whole mushroom chocolate blend) during the second session.

If a participant, or principal investigator, prefers using 20 mg for their second session, or if the principal investigator recommends that a participant repeat the 20-mg dose, this is an acceptable option. To optimize subject safety, all participants will be observed for a minimum of 8 h after dosing, and during that period, a qualified physician with current certification in basic life support will be available to evaluate the participant in person within 15 min in case of any urgent need.

The whole psilocybin mushrooms that will be used in our study will be obtained from the Scottsdale Research Institute, a DEA-approved supplier. The product consists of organically grown *Psilocybe cubensis* mushroom (RTT1121 strain also called JMF-01), which has been milled and then blended into a solid chocolate dosage. The composition of the mushrooms is verified by a qualified laboratory. Psilocybin is approximately 1% of the total dried mushroom weight. Each chocolate corresponds to 10 mg (± 10%) of psilocybin. All four facilitators will be present during each dosing day, with at least two facilitators always present in the treatment room. A fifth person from the research team (who may be a medical provider, therapist, or other IRB-approved research staff member known to the participants) will function in a support capacity. The primary treatment room will include comfortable furnishings that allow participants to recline or lie flat. To encourage an inward-directed experience, each participant will be provided with eyeshades and noise-canceling headphones, through which curated music will be played. The same music will be played through speakers in the room. A second comfortably furnished space is available nearby if needed for additional privacy for individual processing. If it is determined that a participant may benefit from temporary separation from the rest of the group, they will be accompanied by one of the facilitators as well as the support staff member until deemed appropriate to return to the group room.

Facilitators will provide reassurance, support, and redirection only as needed and will avoid directive therapy. Blood pressure and heart rate will be checked at approximately 60 min after ingestion of the psilocybin and at one to 2 h intervals for the first 6 hrs after ingestion and prior to participant release. Rescue medications for symptomatic blood pressure excursions as well as common adverse effects (e.g., nausea, headache) will be available and may be administered after clinical assessment by the medical monitor. At approximately six hours after psilocybin ingestion, each participant will then be invited to participate in a facilitated sharing session.

Art supplies and journaling materials will be provided to encourage individual reflection and expression as the subjective effects of the psilocybin wear off. Approximately 7 to 7.5 h after ingestion of the psilocybin, all participants will complete the post-session assessments (a subset of those are included in Table 3).

**Table 3:** Assessments.

Abbreviation	Instrument
LEC-5	Life events checklist for DSM-5
MINI	Mini international neuropsychiatric interview
CAPS-5(CR)	Clinician-administered PTSD Scale for DSM-5
C-SSRS	Columbia suicide severity rating scale
EQ	Expectancy questionnaire
PCL-5	PTSD checklist for DSM-5
SDS	Sheehan disability Scale
MSPSS	Multidimensional scale of perceived social support
DSS-B	Dissociative symptoms scale–brief
BDI-II	Beck depression inventory-II
HAM-A	Hamilton anxiety rating scale
BPIsf	Brief pain inventory short form
WHO-5	WHO-5: World Health Organization-5 Wellbeing Index (Quality of Life)
TLFB	Timeline follow-back (assess substance use)
MIDS	Moral injury and distress scale
MEQ-30	Mystical experiences questionnaire
CEQ	Challenging experiences questionnaire
EBI	Emotional breakthrough inventory
WCS	Watts connectedness scale
SCS	Social connectedness scale
Qualitative interview	Assessment of appropriateness for group therapy by licensed therapist

Prior to release, each participant will be assessed by a physician to ensure physical and psychological safety and that the effects of psilocybin have resolved. All participants will be provided the contact information for the study physician and the lead facilitator, who may be reached if concerns arise. A family member or friend who has been identified as a support person will pick up the participant to escort them home.

### Integration sessions

A total of three integration visits (one individual and two group-format sessions), each lasting approximately 90 min, will follow each dosing session. The purpose of integration is to process the material and insights occasioned by the psychedelic experience. The facilitators’ role is not to interpret or analyze but rather to use open-ended, non-directive inquiry about the psilocybin session and to invite participants to reflect on the experience and provide their own interpretations. In group sessions, facilitators will use a non-directive approach, modeling compassionate curiosity to elicit discussion about how the experience may have affected participants’ perspectives and/or their relationships to their trauma. Importantly, facilitators also will respond to and manage the dynamics of the group sessions. The three integration sessions will, if possible, be facilitated by the same licensed therapist, with the goal of having all four facilitators present at the group sessions. The third integration session after the first dosing session will include dedicated time to address any participant concerns, revisit intentions, and review safety protocols and consent in anticipation of the second dosing session. The same pattern of group-format and individual integration sessions and scale completion will occur after the second psilocybin session.

Following these integration sessions, three peer-led group visits will be held virtually at four, six, and 10 weeks after the second psilocybin session. These sessions will be designed to provide continued integration support, foster a sense of community, and identify participants who may need additional support. Optional monthly check-in sessions will be held virtually and will be led by the peer-facilitator(s) and/or one of the licensed providers. These will occur at approximately 4, 5, and 6 months after the second psilocybin session. The goals of these sessions are to provide ongoing integration support for up to 6 months as well as fostering a sense of community among participants.

## The circle of care program: a multi-layered and extended integration support model

The Clinical trials and studies in PAT typically include a limited number of integration visits, yet the literature increasingly recognizes that integration is not confined to discrete clinical appointments and healing does not stop when a protocol ends (44). Participants often continue processing relational, behavioral, and existential shifts well after formal protocols conclude. While these experiences may be therapeutic, they can also create a period of increased vulnerability as individuals attempt to reconcile new insights with long-held beliefs about identity, responsibility, and meaning. Ethical analyses of psychedelic research have further emphasized the importance of continuity of care, noting the risks associated with abrupt clinical termination and the corresponding moral obligation to anticipate post-study vulnerability (44).

Our model reflects these concerns identified in the literature and gaps in research design and focus, and incorporates findings from our community engagement process, in which participants consistently identified the need for sustained relational support, practical scaffolding, and continued community connection following the clinical phase. Recognizing that PAT may catalyze changes in values, relationships, spirituality, and life direction, we have designed an extended post-protocol integration program called the Circle of Care to support participants as they navigate these shifts over time.

Abrupt clinical termination in this context is not a neutral event; it is an ethical risk. Extended integration models are not supplemental. They are a prerequisite for psychedelic-assisted care that is safe, equitable, and translatable into real-world public health systems (41). The literature increasingly recognizes that integration is not confined to discrete clinical appointments, but rather unfolds as an iterative, multidimensional process encompassing cognitive, emotional, behavioral, and social dimensions of healing that extends well beyond the acute pharmacological event itself (41). Research on peer support outcomes in veterans with PTSD further suggests that recovery is best understood as an ongoing process of integrating the experience of trauma into daily life and identity, rather than as resolution of symptoms — a conceptualization with direct implications for how post-protocol support is structured (45).

This approach reflects the understanding that healing from trauma — particularly in cases of PTSD and complex PTSD — is often nonlinear and continues beyond the timeframe of a clinical protocol, making extended, participant-directed, and community-based integration options essential not only for participant safety, but for the development of treatment models that are acceptable, sustainable, and scalable in real-world settings.

The Circle of Care is a three-month post-protocol integration program beginning at the conclusion of the 16-week clinical protocol, designed to support continued embodiment and integration following study completion. Like the use of psilocybin, the group treatment format, and the PTSD focus, an extended integration model was a predetermined component of this study — established at the outset and continuously informed by the community engagement process. It extends the integration arc beyond the formal clinical phase into community-based and self-directed support.

The Circle of Care post clinical study integration program has three core components:

1. Two program-covered sessions per month — either individual or group sessions with vetted providers, available to study participants and their networks of support, including family members, partners and loved ones
2. A curated digital resource network mapping local, state, and national support services across clinical and non-clinical domains, designed to serve both participants and the people closest to them-recognizing that PAT can catalyze relational shifts that extend beyond the individual into their most significant relationships
3. A moderated, cohort-based communication channel sustaining peer connection without recreating clinical dependence

Providers included in the program span six domains commonly activated in trauma recovery and PAT: Clinical Mental Health & Therapy; Somatic & Physical Health; Peer Support & Community Connection; Creative & Expressive Arts; Spiritual & Faith-Based Support: and Social Services & Life Skills Support. Listings encompass licensed clinicians, non-clinical practitioners, peer networks, and community organizations, available in-person or virtually. Select listings are also available to participants’ family members and support persons, recognizing that PAT can catalyze relational shifts that extend beyond the individual participant into their closest relationships. Providers will be vetted through a standardized assessment of qualifications and an interview process, with the pilot network anticipated to include between 30 and 50 clinical and non-clinical facilitators and practitioners.

### Development, learning, and future application

The Circle of Care is being developed and coordinated by the PMHA Alliance in deep collaboration with the GPAT research and clinical teams at the University of New Mexico. New Mexico serves as the initial implementation site, with participant feedback and narrative accounts informing iterative refinement as the model is adapted for future jurisdictions. In this way, the local provider network — drawn from practitioners, therapists, and community-based organizations identified through the community co-design process — ensures that the Circle of Care is itself an extension of the community engagement from which this study was born.

### The importance of cost modeling and economic analysis

A major barrier to scaling psychedelic-assisted therapy (PAT) is not only clinical uncertainty but operational uncertainty: what it actually costs to deliver a safe, comprehensive model of care in routine settings, particularly for populations facing structural barriers to access. To address this evidence gap, the UC Berkeley Collaborative for the Economics of Psychedelics (CEP)^1^ will conduct a prospective micro-costing study embedded in the pragmatic, open-label Group Psilocybin-Assisted Therapy (GPAT) pilot for post-traumatic stress disorder (PTSD). The analysis will capture delivery costs for community engagement, preparation, administration, and extended integration supports as implemented in an equity-oriented, community-informed protocol.

The primary analytic perspective will be that of a public payer/health system, aligned with decision needs in New Mexico (e.g., Medicaid and state appropriations). The cost boundary includes the costs of service delivery: personnel time, space, supplies and equipment, training and supervision, and administrative overhead. Because access barriers are often practical rather than clinical, we will also record selected participant-enabling supports (e.g., transportation assistance or childcare during dosing-day visits) that may be necessary for participation but are typically omitted from clinical-trial budgets.

## Methods activity mapping and micro-costing

The Collaborative for the Economics of Psychedelics, in partnership with the PMHA Alliance and UNM leadership, will map the GPAT workflow through document review, structured discussions with site leadership and frontline staff, and site visits. Using micro-costing methods, we will identify each resource required to deliver the model, measure its quantity, and assign a unit cost to estimate total costs. Measurement will emphasize observable delivery activities: staffing time by role (licensed clinicians, physician/medical monitoring, peer facilitators, research coordination, and administration), the “dose” of preparation/dosing/integration delivered, facility use (room time and relevant equipment), training and supervision time, and participant support services. Incomplete or shared resources (e.g., shared staff time, volunteer time, and in-kind contributions) will be documented explicitly and handled via prespecified allocation rules to support transparent sensitivity analysis.

### Cost components

The model will distinguish fixed or semi-fixed start-up costs from variable costs per cohort and per participant. Start-up costs include community engagement and co-design activities; protocol refinement; development of monitoring and documentation systems; and initial training and supervision systems for licensed clinicians and peer facilitators. Variable costs include facilitator time during individual and group preparation sessions; dosing-day facilitation and on-site medical monitoring; post-session safety follow-up; in-person and virtual integration sessions; and community-based supports, as applicable. Because the protocol is designed to be pragmatic, we will also document how costs change when common comorbidities or practical barriers require additional preparation, monitoring, or referral coordination.

### Analytic focus: cost drivers and delivery scenarios

A central aim is to quantify how group format and staffing mix change the cost structure relative to prevailing individual-session PAT models. Prior empirical work suggests (28) that group-format PAT can reduce clinician labor time per patient by approximately 35–50% and reduce overall costs by up to about 20% compared with fully individual delivery, largely through shared preparation and integration time and more efficient deployment of highly trained clinicians. We will translate the GPAT schedule into per-participant clinician-hours (and peer-hours) by phase, then examine plausible delivery scenarios relevant to scalability and workforce constraints: cohort size, number and duration of preparation and integration sessions, role substitution (peer-led vs. clinician-led elements), and the use of virtual platforms for portions of integration. Scenario analyses will be exploratory, intended to identify the levers most likely to affect cost and access, rather than to “optimize” the program prematurely.

### Outputs and linkage to future value assessment

The Collaborative for the Economics of Psychedelics will deliver (i) a standardized costing instrument intended to be reusable across community-based PAT sites; (ii) descriptive estimates of total and phase-specific cost per participant and cost by resource category; and (iii) payer-relevant scenario results to inform program budgeting and reimbursement planning. At this stage, the economic component is limited to cost analysis rather than cost-effectiveness analysis; its purpose is to generate rigorous delivery-cost estimates and costing methods that will provide the foundation for future cost-effectiveness work as stronger comparative clinical outcome data become available. As clinical and implementation outcomes accrue, these cost estimates can be paired with outcomes collected in the study (e.g., changes in PTSD symptoms, functioning, quality of life, and participant acceptability) to support preliminary cost-and outcome-based comparisons relative to standard care pathways. Given the pilot’s limited sample size and open-label design, we will be cautious about definitive cost-effectiveness conclusions; instead, we will emphasize transparent cost estimates, uncertainty ranges, and clear specification of assumptions.

### Limitations and mitigation

Community-based costing is vulnerable to incomplete capture and workflow variation across cohorts. We will mitigate these risks by using standardized tracking tools for staff time and resource use, incorporating verification steps with site leadership, conducting periodic review and validation of cost data during the pilot, and maintaining clear documentation so results can be recalculated under alternative costing conventions if needed.

## Conclusion

Here we present a description of a pragmatic open-label clinical trial protocol for group psilocybin-assisted therapy for PTSD that is the first PAT study developed in alignment with a community engagement process. The study is also novel in the use of a whole psilocybin IMP, peer as well as licensed therapist facilitators, and an extended integration process. These factors were intentionally incorporated to address concerns related to accessibility, pragmatic inclusion and exclusion criteria, cultural appropriateness, and post-dosing support. Although this study is not designed to compare this approach with existing experimental approaches in the literature, we believe that the incorporation of these factors not only will enhance acceptance of PAT but also may improve response rates and longer-term outcomes. As New Mexico is initiating a state regulated model for the therapeutic use of whole psilocybin mushrooms the study is being performed concurrently with a micro-costing process to assess delivery costs. We hope that our data relating to feasibility, cost, and clinical outcomes will help inform the structure of the New Mexico Medical Psilocybin program as well as emerging PAT programs across the United States and beyond.

## Footnotes

1
https://cghdde.berkeley.edu/projects/collaborative-economics-psychedelics-cep
